# Metabolomics Analysis Reveals Global Metabolic Changes in the Evolved *E. coli* Strain with Improved Growth and 1-Butanol Production in Minimal Medium

**DOI:** 10.3390/metabo10050192

**Published:** 2020-05-13

**Authors:** Walter A. Laviña, Sana Subhan Memon Sakurai, Sammy Pontrelli, Sastia Prama Putri, Eiichiro Fukusaki

**Affiliations:** 1 Microbiology Division, Institute of Biological Sciences, University of the Philippines Los Baños, Los Baños, Laguna 4031, Philippines; walavina@up.edu.ph; 2 Department of Biotechnology, Graduate School of Engineering, Osaka University, 2-1 Yamadaoka, Suita, Osaka 565-0871, Japan; sanamsakurai@gmail.com (S.S.M.S.); sastia_putri@bio.eng.osaka-u.ac.jp (S.P.P.); 3 Institute of Molecular Systems Biology, D-BIOL, ETH Zurich, 8092 Zurich, Switzerland; pontrelli@imsb.biol.ethz.ch

**Keywords:** 1-butanol production, adaptive laboratory evolution, *Escherichia coli*, metabolomics, minimal medium

## Abstract

Production of 1-butanol from microorganisms has garnered significant interest due to its prospect as a drop-in biofuel and precursor for a variety of commercially relevant chemicals. Previously, high 1-butanol titer has been reported in *Escherichia coli* strain JCL166, which contains a modified *clostridial* 1-butanol pathway. Although conventional and metabolomics-based strain improvement strategies of *E. coli* strain JCL166 have been successful in improving production in rich medium, 1-butanol titer was severely limited in minimal medium. To further improve growth and consequently 1-butanol production in minimal medium, adaptive laboratory evolution (ALE) using *mutD5* mutator plasmid was done on JCL166. Comparative metabolomics analysis of JCL166 and BP1 revealed global perturbations in the evolved strain BP1 compared to JCL166 (44 out of 64 metabolites), encompassing major metabolic pathways such as glycolysis, nucleotide biosynthesis, and CoA-related processes. Collectively, these metabolic changes in BP1 result in improved growth and, consequently, 1-butanol production in minimal medium. Furthermore, we found that the mutation in *ihfB* caused by ALE had a significant effect on the metabolome profile of the evolved strain. This study demonstrates how metabolomics was utilized for characterization of ALE-developed strains to understand the overall effect of mutations acquired through evolution.

## 1. Introduction

**Figure 1:**
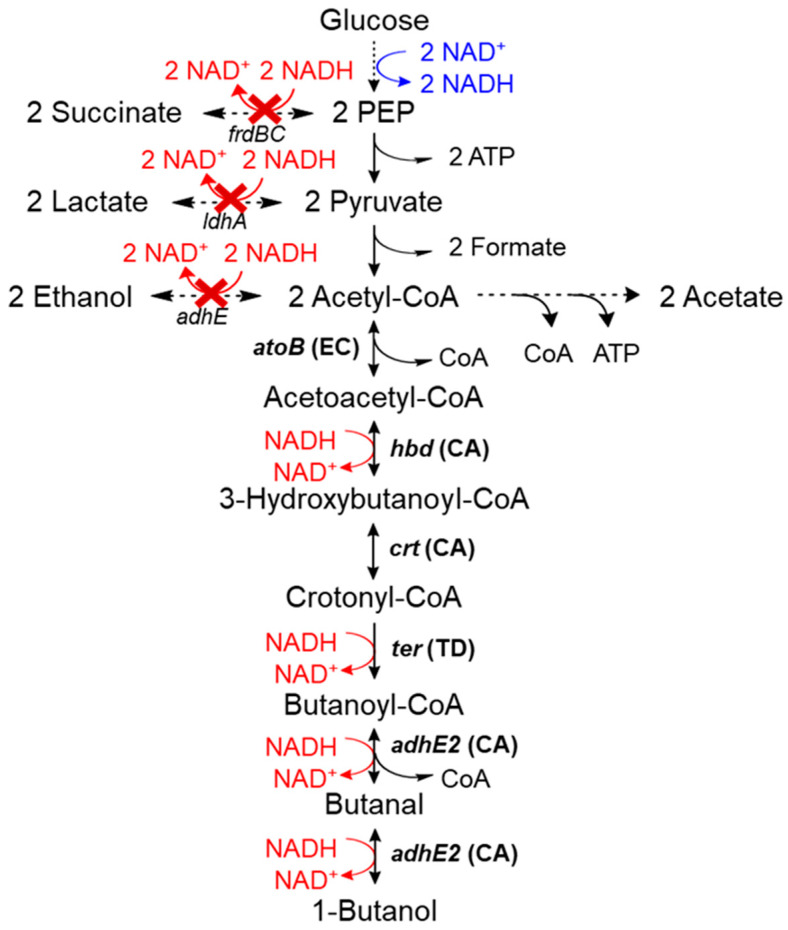
*Clostridial* 1-butanol pathway in *E. coli* carried by two plasmids: pIM8 (*ter*_TD_) and pEL11 (*atoB*_EC_-*adhE2*_CA_-*crt*_CA_-*hbd*_CA_). NADH driving force was established by deletion of genes involved in mixed-acid fermentation reaction (lactate, ethanol and succinate). The resulting strain is called JCL166 (JCL16 *∆ldhA ∆adhE ∆frdBC*).

Production of 1-butanol from microorganisms through the acetone–butanol–ethanol (ABE) fermentation pathway, as exemplified in *Clostridium* sp., has attracted interest due to its potential as alternative fuel and chemical feedstock [1]. To address this, a modified CoA-dependent 1-butanol pathway from *Clostridium acetobutylicum,* carried on plasmids pEL11 and pIM8, has been successfully reconstructed in *Escherichia coli* strain JCL166 (Table 1) [2,3]. To create a driving force for 1-butanol production, major NADH-consuming fermentation pathways were removed by deleting genes for fumarate reductase (*frdBC*), lactate dehydrogenase (*ldhA*) and alcohol dehydrogenase (*adhE*). Under these circumstances, the 1-butanol pathway serves as the sole electron sink for regeneration of NAD^+^. Therefore, growth and 1-butanol production were coupled in strain JCL166 under anaerobic conditions, wherein high 1-butanol production has been achieved (Figure 1) [4,5,6].

In the high-producing strain JCL166, the intracellular NADH driving force was further increased by overexpression of formate dehydrogenase (*Fdh*) from *Candida biodinii,* which catalyzes oxidation of formate to CO_2_ and NADH [3]. Further modifications include deletion of *pta* (encoding phosphate acetyltransferase) to enhance the acetyl-CoA driving force [3], optimization of AdhE2 and AtoB enzyme activities by improving the RBS translation initiation rate [4,6] and deletion of *aceA* (encoding isocitrate lyase) to decrease acetate accumulation (Figure 1) [5]. However, all the reported increase in 1-butanol titers of the modified host strain JCL166 were completely dependent on growth in rich medium. When minimal medium is used, growth and 1-butanol production were severely restricted even with the aforementioned improvements in the strains.

In the fermentation industry, the use of complex media poses several major disadvantages such as being prone to significant lot-to-lot variations, which results in inconsistent and unstable fermentation performance [8,9]. Thus, the use of chemically defined or minimal media is more applicable for commercial fermentation as it ensures consistency among fermentation batches and, hence, would allow for efficient quality control. Furthermore, minimal media are less sensitive to sterilization conditions and permit a more simplified downstream processing [8,9]. For these reasons, improvement in the performance of the 1-butanol-producing *E. coli* strain in minimal media is required.

Adaptive laboratory evolution (ALE) or directed strain evolution has become a widely used tool in metabolic engineering for generating a variety of industrially relevant strains. In microbial ALE, microorganisms are exposed to prolonged culturing steps in a controlled environment with a continuous selection pressure. Culturing under selection pressure allows for the accumulation of beneficial mutations that result in the improvement of fitness of the strain. ALE has been increasingly used to enhance tolerance of microbial strains to various growth-inhibiting conditions [10,11,12,13] and to improve growth rates in various medium compositions [14,15,16]. 

ALE can be particularly useful in restoring impaired physiological processes essential for growth that often accompany extensive modifications in engineered strains such as the previously constructed 1-butanol-producing strain of *E. coli*, JCL166 [3,7]. In particular, genetic modifications undertaken to improve the production of 1-butanol in *E. coli* strain JCL166 also resulted in physiological changes that restricted growth in minimal media. Reverting these impaired processes by rational design approaches is not a practical route as the targets are often non-obvious and modifications are time-consuming. Hence, ALE can provide a more effective and efficient solution.

For improvement of growth and 1-butanol production of the engineered *E. coli* in minimal medium, ALE using a *mutD5* mutator plasmid was performed on JCL166 [7]. A metabolomics-based approach was utilized to identify potentially important metabolites that were perturbed after performing ALE in JCL166. Comparative metabolomics was done by comparing the parental strain JCL166 to the evolved strain BP1. From LC/MS/MS analysis, nearly 70% of the annotated metabolites were significantly different between the two strains. Moreover, it revealed global perturbations in the evolved strain encompassing major metabolic pathways such as glycolysis, nucleotide biosynthesis, and CoA-related processes. Together, these metabolic changes in BP1 resulted in improved growth and, consequently, 1-butanol production in minimal medium. 

In this study, metabolomics was found to be useful in characterizing ALE-developed strains and the effectiveness of metabolomics for understanding the overall effect of mutations acquired through evolution was proven. The metabolomics-based strategy, coupled with other omics methods, will be useful in accelerating the process of characterizing metabolic and phenotypic changes in evolved strains for the next round of strain improvement experiments and optimization of production conditions for 1-butanol in minimal medium.

## 2. Results and Discussion 

### 2.1. ALE Resulted in Global Metabolic Changes That Enhanced Growth and 1-Butanol Production in E. coli Strain BP1

**Figure 2:**
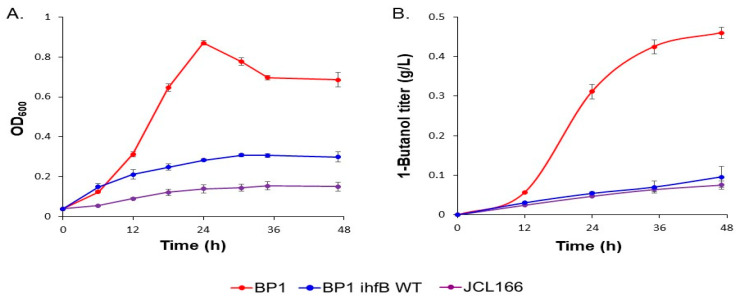
(**A**) Growth curve of JCL166, BP1, and BP1 ihfB WT strain in 4-g/L glucose minimal medium under anaerobic conditions. (**B**) 1-Butanol titer (g/L) of JCL166, BP1, and BP1 ihfB WT strain from anaerobic fermentation in 4-g/L minimal medium. Error bars indicate the standard deviation obtained from three biological replicates.

Although JCL166 has been reported to produce 6.5 g/L of 1-butanol in rich medium after 72 h [3], the strain is unable to produce 1-butanol in minimal medium [7]. Previously, ALE was performed in *E. coli* strain JCL166 in an attempt to increase 1-butanol production. Briefly, the parental *E. coli* strain JCL166 was transformed with plasmid pALQ32 containing *mutD5* and grown in LB at 37 °C overnight. Successive serial inoculations of the culture were done in M9 minimal media with a decreasing amount of LB medium supplementation. After evolution of JCL166 and curing of the mutator plasmid, the resulting clonal strains were screened for growth in minimal medium and ability to produce 1-butanol [7]. Our previous result showed that, after 30 serial dilutions, ALE was able to substantially restore growth in the evolved strain and, since growth is coupled with production in this strain, it allowed for an increased 1-butanol production in minimal media (Figure 2A,B). Since the metabolome is a reflection of the cell’s genome-encoded enzymatic pathways and networks, metabolomics can provide a snapshot of the actual physiological state of the cell [17]. Thus, metabolic profiling was carried out at an early stationary phase (24 h) when the difference in 1-butanol production was substantial (Figure 2B). In this case, the physiological states of the *E. coli* parental strain JCL166 and evolved strain with the highest 1-butanol production among the clonal strains screened, BP1, were compared.

### 2.2. Comparative Metabolic Profiling of Parental Strain (JC166) and Evolved Strain (BP1) Revealed Differences in Intracellular Levels of Several Metabolite Groups 

Ion-pair triple quadrupole liquid chromatography mass spectrometry (IP-LC/QqQ-MS) detected a total of 64 metabolites in JCL166 and BP1 strains during stationary phase [7]. The annotated metabolites were subjected to principal component analysis (PCA) in which PC1, accounting for 68.9% of the total sample variance, showed clear separation of the two strains (Figure S1A). After examining the loading plot for metabolites that contributed to the separation, we found a significant number of metabolites (44 out of 64 (*p* ≤ 0.05)) that were perturbed after evolution (Figure S1B,C). [7]. This difference suggests that laboratory evolution of *E. coli* with heterologous expression of 1-butanol pathway enzymes resulted in global metabolic changes that provoke optimal glucose consumption, growth and 1-butanol production in minimal medium. The global effect of ALE on the metabolome profile suggests the presence of mutations in genes encoding for proteins that may elicit a system-wide response such as genes for global regulation of transcription.

The metabolites contributing most to the separation of the strains in PC1 included CoA-related metabolites such as acetyl-CoA and butanoyl-CoA (Figure S1B), wherein they accumulated more in BP1 as compared to JCL166 (Figure S2). Moreover, BP1 had lower relative intensity of free CoA (Figure S2), suggesting that free CoA was utilized more efficiently within the 1-butanol pathway. Pantothenate, a CoA precursor, was also found in higher relative intensity in BP1 (Figure S2). The increased pantothenate level in BP1 suggests that adaptive evolution resulted in the improved ability of the cell to supply CoA for the 1-butanol production pathway.

Metabolites that contributed most to the separation in PC1 also included glycolytic intermediates dihydroxyacetone phosphate (DHAP), fructose-6-phosphate (F6P), 2-phosphoglyceric acid (2PGA) and 3-phosphoglyceric acid (3PGA) (Figure S1C). Along with phosphoenolpyruvate (PEP), these annotated glycolytic metabolites were observed in significantly lower relative intensities in the BP1 strain compared to JCL166 (Figure S3). The decreased accumulation after evolution may suggest improved glycolytic enzymatic activities that resulted in faster conversions. Indeed, glycolytic enzymatic activities in BP1 strain were shown to increase as a result of adaptive evolution [7] Additionally, glucose uptake was increased almost 5-fold after evolution, with BP1 consuming 3.31 ± 0.04 g/L glucose compared to 0.68 ± 0.18 g/L that was consumed by JCL166 after 24 h of anaerobic growth. Improved glycolysis and increased glucose uptake most likely allowed for improved growth and, consequently, 1-butanol production in the evolved strain, BP1.

Annotated nucleosides such as guanosine, thymidine, uridine and cytidine were all found to accumulate in JCL166 (Figure S1C and S4). On the other hand, these metabolites were either not detected or present in significantly lower relative intensities in BPI compared to JCL166 (Figure S4). The decreased nucleosides in BP1 could suggest an increased use of these metabolites for nucleotide biosynthesis. In addition to these metabolites, phosphoribosyl pyrophosphate (PRPP), a key metabolite in the biosynthesis of purine and pyrimidine nucleotides, was also observed in lower relative intensity in BP1 (Figure S1C and S4). Furthermore, amino acids used in nucleotide synthesis such as glutamine and aspartate were also found to be significantly lower in BP1 (Figure S1C and S4).

### 2.3. Comparative Metabolic Profiling of BP1 and Wild-Type Reversion Strain BP1 ihfB WT

#### 2.3.1. Principal Component Analysis of BP1 and BP1 *ihfB* WT Reveals That Mutation in *ihfB* Has a Large Effect on the Metabolome Profile

Metabolomics analysis revealed that ALE of the parental strain JCL166 resulted in global metabolic changes in the evolved BP1 strain. Whole genome sequencing to determine the total mutations acquired by BP1 after ALE revealed 113 mutations that included a nonsynonymous point mutation in *ihfB*, the gene encoding for one of the two subunits of the integration host factor (IHF) [7]. Since mutations in *ihfB* are expected to have a large effect on the metabolome of the cell as IHF is a key global regulator of transcription [18,19], this specific mutation was further investigated in the study. To determine the significance of the *ihfB* mutation in the acquired phenotype of BP1, reversion of the point mutation to the wild-type sequence was done, producing strain BP1 *ihfB* WT [7]. In this study, we performed comparative metabolomics analysis of BP1 and the wild-type reversion strain of the *ihfB* mutation, BP1 *ihfB* WT. Reversion to the wild-type sequence in BP1 *ihfB* WT resulted in decreased growth and 1-butanol production (Figure 2) to a level that is similar to the parental strain JCL166. This indicates the importance of the *ihfB* mutation in the improved growth and 1-butanol production of BP1 in minimal media. On the other hand, although it is possible that the introduction of *ihfB* mutation could improve production of 1-butanol in JCL166, our previously published report from which BP1 originated has pinpointed several mutations that are essential to the evolved phenotype. Therefore, the contribution of this mutation on the unevolved JCL166 would likely provide a negligible effect in improving 1-butanol titers [7].

**Figure 3:**
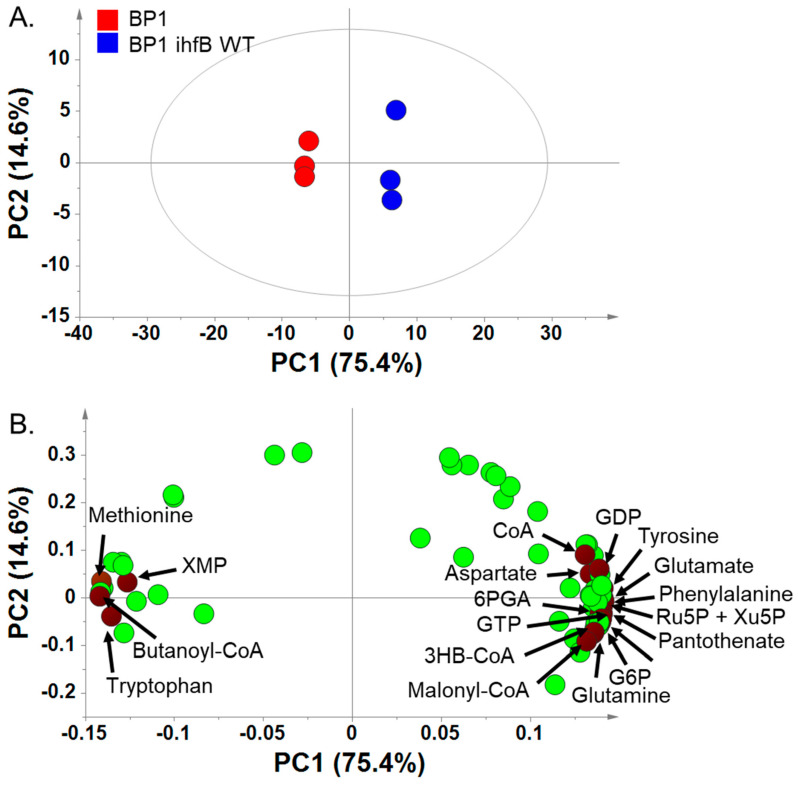
(**A**) PCA score plot for metabolic profiling of BP1 and BP1 *ihfB* WT at mid-log phase. The ellipse indicates 95% confidence border based on Hotelling’s T^2^. (**B**) Corresponding PCA loading plot showing metabolites that contributed to the separation of the two strains.

Although the phenotype of BP1 *ihfB* WT was more similar to JCL166 than to BP1, comparison of the revertant strain with JCL166 cannot provide insights regarding the effect of the acquired mutation. This is due to the presence of a large number of genetic differences between JCL166 and BP1 *ihfB* WT. Hence, to ascertain the effect of the acquired mutation in *ihfB* gene on the metabolic state of BP1, comparison of metabolome profiles between BP1 and BP1 *ihfB* WT was carried out. Metabolic profiling of BP1 and BP1 *ihfB* WT was done at mid-log phase, when the cell is most active. Using IP-LC/QqQ-MS, 66 metabolites were annotated in both strains at mid-log phase (Table S1), and were subjected to PCA. PC1, accounting for 75.4% of the total sample variance, separated the two strains clearly (Figure 3A). The loading plot was examined for metabolites that contribute to the separation in PC1 (Figure 3B). Most of the annotated metabolites contributed positively, accumulating in BP1 *ihfB* WT with higher relative intensities compared to the BP1 strain.

#### 2.3.2. Mutation of *ihfB* in BP1 Affects Intracellular Amino Acid Levels

**Figure 4:**
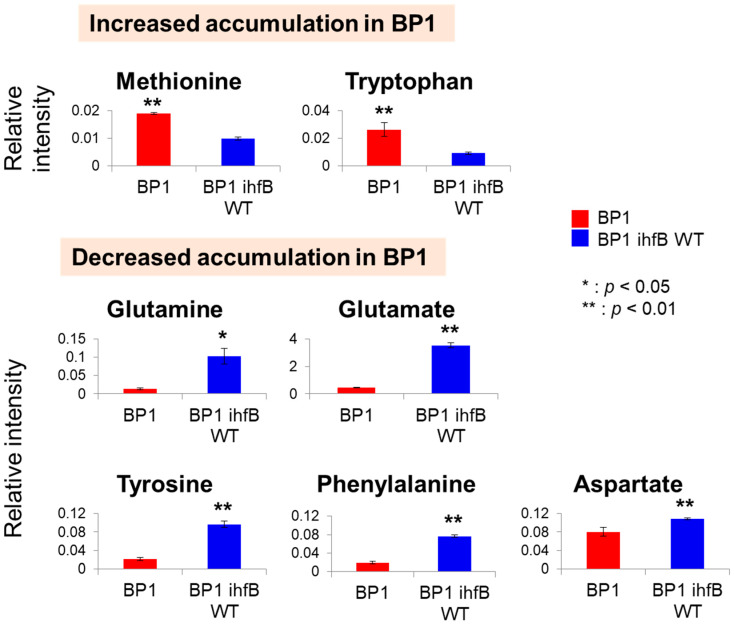
Metabolite intensities of amino acids. Bar graphs show relative intensity on y-axis obtained by normalization of peak area with the internal standard. Asterisks indicate significant differences among the two strains (*: *p* < 0.05, **: *p* < 0.01). Error bars indicate standard deviation obtained from three replicates.

**Figure 5:**
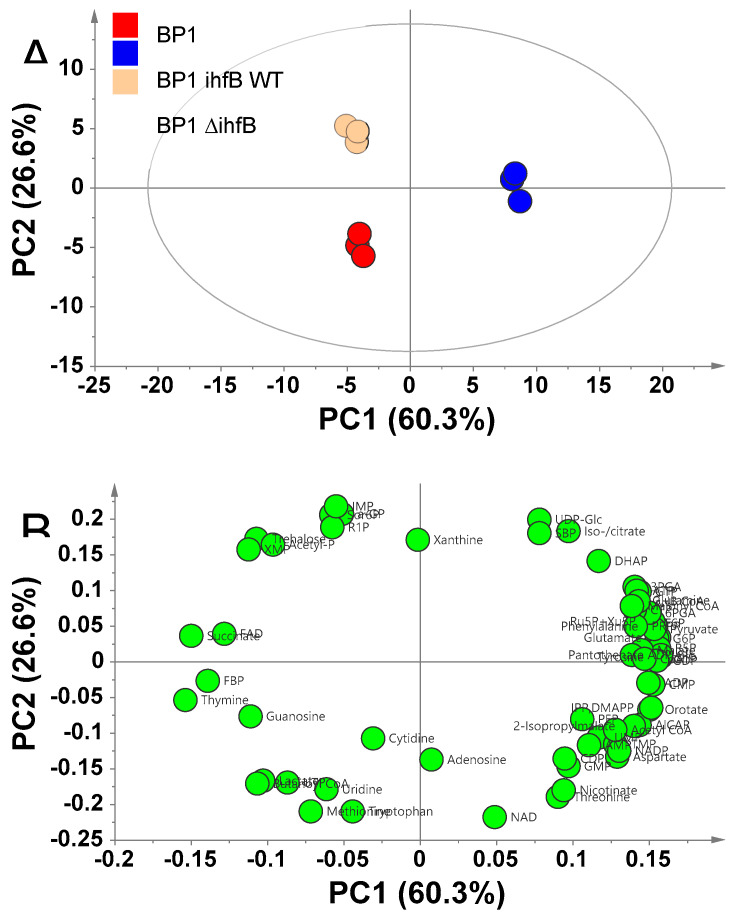
(**A**) PCA score plot for metabolic profiling of BP1, BP1 *ihfB* WT and BP1 *∆ihfB* at mid-log phase. The ellipse indicates a 95% confidence border based on Hotelling’s T2. (**B**) Corresponding PCA loading plot showing metabolites that contributed to the separation of the three strains.

Upon mutation of *ihfB* in BP1, methionine and tryptophan levels were increased while glutamine, glutamate, tyrosine, phenylalanine and aspartate were decreased (Figure 4). IHF has been shown to regulate genes involved in amino acid biosynthesis by decreasing *glnA* and *gltB* gene expression [20,21] that are involved in glutamine and glutamate biosynthesis, respectively. Furthermore, an increase in *thrA* gene expression that is involved in the biosynthesis of the ‘aspartate family’ of amino acids (lysine, methionine and threonine) has also been observed in the IHF mutant [20]. Assuming that mutation in *ihfB* in BP1 strain results in a similar phenotype to IHF deletion mutants, decreased *glnA* and *gltB* expression would possibly result in lower glutamine and glutamate concentrations. This was in fact the case for the BP1 strain. In addition to this, increased *thrA* expression could possibly result in lower aspartate levels and higher level of ‘aspartate family’ of amino acids. To confirm if IHF function in BP1 strain was lost, *ihfB* was deleted from the BP1 strain to produce strain BP1 Δ*ihfB* [7]. Indeed, metabolome profile of BP1 strain was found to correlate more closely to BP1 *∆ihfB* strain than to BP1 *ihfB* WT (Figure 5). In terms of growth and 1-butanol production, BP1 Δ*ihfB* and BP1 *ihfB* WT similarly showed a decrease as compared to BP1 (Figure S5). This result indicates that mutations in *ihfB* confer a beneficial effect on the strain without completely inhibiting enzyme function.

#### 2.3.3. Mutation of *ihfB* in BP1 Affects Intracellular Levels of the Pentose Phosphate Pathway and GTP Biosynthesis Metabolites

**Figure 6:**
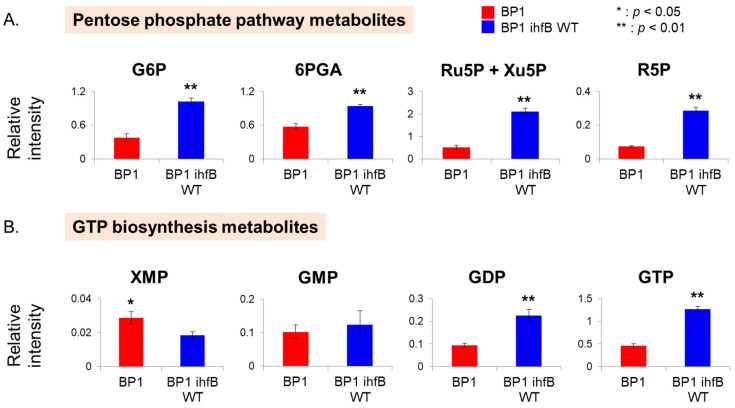
(**A**) Metabolite intensities of pentose phosphate pathway intermediates. (**B**) Metabolite intensities of XMP and guanosine phosphates. Bar graphs show relative intensity on y-axis obtained by normalization of peak area with the internal standard. Asterisks indicate significant difference amongst the two strains (*: *p* < 0.05, **: *p* < 0.01). Error bars indicates standard deviation obtained from three replicates.

From the PCA plot, pentose phosphate pathway (PPP) intermediates were shown to contribute to the separation of BP1 from BP1 *ihfB* WT in PC1 (Figure 3B). Detected PPP metabolites, glucose 6-phosphate (G6P), 6-phosphogluconate (6PGA), ribulose 5-phosphate (Ru5P), xylulose 5-phosphate (Xu5P) and ribose 5-phosphate (R5P), were all found in lower relative intensities in BP1 strain compared to the wild-type reversion strain, BP1 *ihfB* WT (Figure 6A). Under anaerobic conditions, flux into PPP and citric acid cycle (TCA) is low [22]. Thus, lower relative intensities of PPP pathway metabolites may suggest the further reduction of carbon source entering PPP upon mutation in *ihfB* gene. Although the relationship between IHF and PPP is not clear, IHF is known to co-regulate genes along with several other transcription factors (e.g., CRP, FNR, ArcA, FIS, Hns, Lrp) [19], some of which are related to PPP (e.g., FNR) [23]. 

Guanosine diphosphate (GDP) and guanosine triphosphate (GTP) were also found to contribute to the separation in PC1 (Figure 3B). The relative intensities of GDP and GTP were significantly higher after the reversion of point mutation acquired from ALE (Figure 6B). In addition, xanthosine monophosphate (XMP), the precursor of purine nucleotides, was found on the negative side of PC1 (Figure 3B). The increased relative intensity of XMP and decreased amount of GDP and GTP in BP1 strain compared to BP1 *ihfB* WT (Figure 6B) may suggest a decreased GTP biosynthesis after mutation in the *ihfB* gene. The *E. coli* IHF mutant has been shown to have lower *guaA* gene expression than the wild type. The *guaA* gene encodes for GMP synthetase, which is responsible for the conversion of XMP to GMP [20]. 

#### 2.3.4. Mutation of *ihfB* in BP1 Affects Intracellular Levels of CoA-Related Metabolites

**Figure 7:**
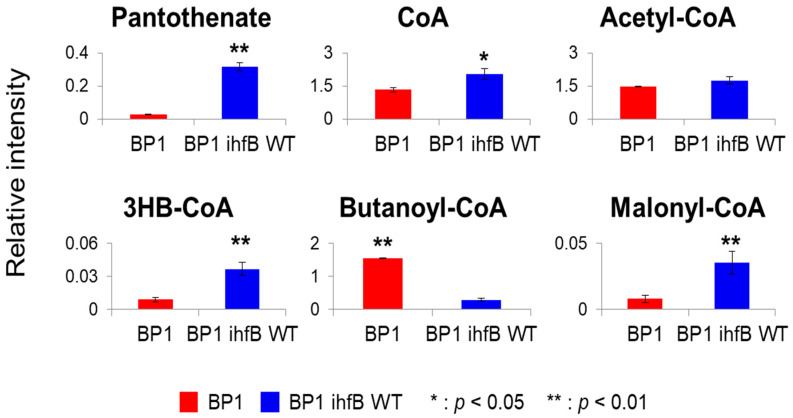
Metabolite intensities of CoA-related compounds. Bar graphs show relative intensity on y-axis obtained by normalization of peak area with that of internal standard. Metabolite levels with significant differences between the two strains are marked with asterisks (*: *p* < 0.05, **: *p* < 0.01). Error bars indicate standard deviation obtained from 3 biological replicates.

Pantothenate was one of the metabolites contributing most to the separation of strains in PC1 wherein it was found to be significantly higher in BP1 *ihfB* WT (Figure 3B; Figure 7). Reduced level in BP1 could suggest a more efficient use of this precursor for CoA production. However, the relative intensity of CoA was also decreased in the BP1 strain upon mutation in the *ihfB* gene (Figure 7). This could suggest that CoA production is lower when mutation in the *ihfB* gene is present. Alternatively, it could also suggest that CoA is being used up for the 1-butanol pathway. The latter explanation is supported by an increased relative intensity of butanoyl-CoA in BP1 (Figure 7). 

Interestingly, the level of 3-hydroxybutanoyl-CoA (3HB-CoA) was higher after the mutation was reverted back to wild-type (Figure 7). Higher 3HB-CoA levels in BP1 *ihfB* WT could suggest that mutation in *ihfB* gene in BP1 allowed better channeling of 1-butanol pathway intermediates for 1-butanol production. Although, intracellular acetyl-CoA levels were comparable between the two strains, an increased intensity of malonyl-CoA was observed in BP1 *ihfB* WT (Figure 7), which may suggest that CoA and acetyl-CoA are channeled into synthesis of fatty acids instead of 1-butanol production when the mutation in *ihfB* is reverted back to wild-type.

## 3. Materials and Methods 

### 3.1. E. coli Strains and Plasmids

*E. coli* strains and plasmids used in this study are summarized in Table 1.

### 3.2. Medium and Growth Conditions

For the pre-culture, strains were cultivated in 8 mL of LB medium with appropriate antibiotics (100 µg/mL ampicillin and 50 µg/mL kanamycin) in 50 mL Falcon tubes incubated at 200 rpm for 17 hours at 37 °C. Cells were harvested by centrifugation and washed twice with 8 mL of minimal medium before inoculation into the main culture. Thirty milliliters (30 mL) of minimal medium (commercial 5× M9 salts supplemented with final concentrations of 4 g/L glucose, 1 mM MgSO_4_, 0.1 mM CaCl_2_ and 1 µg/mL thiamine (vitamin B1)) containing appropriate antibiotics and 0.1 mM isopropyl-β-D-thiogalactopyranoside (IPTG) was inoculated with an initial OD_600_ of 0.04. The cultures were placed in 100-mL Maruemu vial bottles with rubber stoppers and aluminum crimp caps. Immediately after inoculation, the culture condition was made anaerobic by removing the oxygen in the culture tube through a needle (20 G by 1 ½ in.; Terumo, Japan), with the end attached to a 0.22-µm PES (polyethersulfone) filter (Millipore, MA, USA). Repeated vacuuming and refilling of nitrogen gas in an anaerobic chamber were performed. After the needles were removed from the rubber caps in the anaerobic chamber, the flasks were incubated at 37 °C in 200-rpm air shakers. 

### 3.3. Metabolome Sampling and Extraction

Intracellular samples equivalent to 3 OD_600_ units were collected by fast filtration using a 47-mm diameter nylon membrane with a pore size of 0.45 µm (Millipore, MA, USA). The 2-mL sampling tubes containing the filter with cells were immediately quenched by immersing in liquid nitrogen. Sampling was done in triplicates. Samples were stored in −80 °C until extraction. 

Extraction was carried out by adding 1.8 mL of mixed solvent (methanol/water/chloroform = 5:2:2 *v/v/v*) with 25 μg/L of (+)-10 camphorsulfonic acid to the filter. Camphorsulfonic acid was used as an internal standard for relative quantification using LC/MS/MS. After one hour of incubation at −30 °C, 1050 µL of the mixture was transferred to a new tube containing 525 µL of ultrapure water. After vortexing, polar and non-polar phases were separated by centrifugation at 10,000 rpm for 5 min at 4 °C. Using syringe filtration (0.2 µm PTFE hydrophilic membrane, Millipore, MA, USA), 700 µL of the upper polar phase was transferred to a new tube. Samples were concentrated by centrifugal concentration for approximately two hours and later lyophilized by freeze drying overnight. Samples were stored at −80 °C until analysis.

Extracellular samples were collected from the culture supernatant by centrifugation at 16,000× *g* for 5 min at 4 °C. Samples were stored at −30 °C until analysis. Prior to analysis, the samples were filtered via syringe using a 0.2-µm PTFE hydrophilic membrane (Millipore, MA, USA). 

### 3.4. Analysis of Intracellular Metabolites

Lyophilized samples were dissolved in 50 µL of ultrapure water for ion-pair liquid chromatography mass spectrometry (IP-LC/QqQ-MS) analysis. IP-LC/QqQ-MS analysis was carried out using a Shimadzu Nexera UHPLC system coupled with LCMS 8030 Plus (Shimadzu Corp., Kyoto, Japan) with a CERI (Chemicals Evaluation and Research Institute, Tokyo, Japan) L-column 2 ODS (150 mm × 2.1 mm, particle size 3 µm). The mobile phase (A) was 10-mM tributylamine and 15-mM acetic acid in ultrapure water and mobile phase (B) was methanol. The column oven temperature and flow rate were set at 45 °C and 0.2 mL/min, respectively. The concentration of mobile phase (B) was increased from 0% to 15% from 1.0 to 1.5 min, 15% to 50% from 3.0 to 8.0 min and 50% to 100% from 8.0 to 10.0 min, and held until 11.5 min. The concentration was then decreased to 0% and held until 17 min. The injection volume was 3 µL. Negative ion mode was used for mass analysis. The desolvation line temperature and heat block temperature were 250 °C and 400 °C, respectively. The nebulizer gas flow and drying gas flow were 2 L/min and 15 L/min, respectively. Multiple reaction monitoring (MRM) was used for analysis. Optimized MRM parameters are given in Supplementary Information (Table S2). The raw data was converted to analysis base file (.abf) format using a freely available file format converter (Reifycs Inc., Tokyo, Japan, https://www.reifycs.com/AbfConverter/). Peak picking and peak area calculation were carried out using MRMPROBS [24]. The peak area of each metabolite was normalized using the peak area of (+)-10 camphorsulfonic acid. 

### 3.5. Multivariate Analysis

SIMCA-*p*+ version 13 (Umetrics, Umeå, Sweden) was used for principal component analysis (PCA). Metabolome data was mean centered to unit variance and transformation was not performed. Data matrices subjected to PCA are given in Supplementary Information (Table S1).

### 3.6. Analysis of Extracellular Metabolites

1-Butanol was quantified using a GC-2010 system (Shimadzu, Kyoto, Japan) equipped with a flame ionization detector. InertCapTM Pure-WAX capillary column 0.25 mm I.D. × 30 m, df = 0.25 µm (GL Sciences Inc., Tokyo, Japan) was used. Isobutanol was used as an internal standard. Supernatant culture was diluted with isobutanol and the sample was then injected in split mode with split ratio of 1:15. The injection temperature was maintained at 225 °C. The initial temperature of column was held at 50 °C for 1 min and then raised at a rate of 7 °C/min to 80 °C and held for 1 min. It was then raised with a gradient of 20 °C/min until 120 °C, held for 2 min and then again, raised with a gradient of 50 °C/min to 250 °C and held for 5 min. Nitrogen was used as carrier gas with a column flow rate of 2.21 mL/min (linear velocity of 45.2 cm/s). The inlet pressure was 134.9 kPa. 

The remaining glucose in the culture medium was measured using F-kit D-glucose (Roche Diagnostics, Manheim, Germany) following the manufacturer’s instructions. 

### 3.7. Construction of Wild-Type Reversion Strains

Deletion of *ihfB* gene was performed by using λ-Red DNA recombination method described previously [25]. BP1 with pKD46 [25] was transformed with a kanamycin fragment that has homology to *ihfB* at both ends. Wild-type reversion strains (BP1 *ihfB* WT, BP1 *arcB* WT and BP1 *pcnB* WT) were also generated by linear DNA recombination. Fragments of wild-type genes including the site of point mutation in BP1 were amplified from BW25113 [25]. The resulting amplified sequence flanking a kanamycin resistance cassette around 100 base pairs downstream of the gene was used to transform BP1 carrying pKD46 [25]. The deleted and wild-type reverted fragments were verified by Sanger Sequencing.

## 4. Conclusions

ALE of 1-butanol-producing *E. coli* in minimal media resulted in better growth and production profiles, a trait that was not shown prior to evolution. The evolved strain BP1 showed approximately 5–7 times higher glucose consumption, growth and 1-butanol production after 24 hours of anaerobic growth in minimal media compared to its parental strain, JCL166. The distinction between BP1 and JCL166 strains at the stationary phase was based mainly on metabolite differences in (i) CoA-dependent 1-butanol pathway, (ii) central carbon metabolism and (iii) nucleotide metabolism. Out of the 64 annotated metabolites, 44 metabolites were significantly perturbed after evolution. This suggests that ALE of *E. coli* expressing heterologous enzymes for 1-butanol production resulted in global metabolic changes in minimal media.

Genomic analysis revealed the presence of mutation in *ihfB*, encoding for one of the two subunits of a DNA-binding global transcriptional regulator. Mutation in this global regulator may explain the wide differences found between the metabolome of BP1 and JCL166. Reversion of the acquired point mutation in *ihfB* gene back to the wild-type sequence resulted in a strain (BP1 *ihfB* WT) that showed significantly reduced growth and 1-butanol production. Loss of the improved phenotype from ALE confirms that the mutation in *ihfB* is important in BP1. Metabolome profile of BP1 was compared with BP1 *ihfB* WT to realize the effect of this mutation in BP1 strain. The *ihfB* mutation affected (i) amino acid profiles, (ii) PPP metabolites (iii) nucleotide biosynthesis metabolites and (iv) CoA-related metabolites. 

In conclusion, our results serve as an example of the complex system-wide alterations that underlie phenotypic improvement for engineered strains used for bio-production. Alterations induced with ALE were understood in depth by employing a comprehensive metabolomics strategy. The utility of metabolomics to understand the effect of overall mutations acquired after evolution and the effect of a single important mutation was proven through this study. 

## Figures and Tables

**Table 1:** Strains and plasmids used in this study. Subscripts in plasmids indicate the source of the gene: EC—*Escherichia coli*; CA—*Clostridium acetobutylicum*; TD—*Treponema denticola.*

Strains	Description or Genotype	Source
JCL166	BW25113/F’ [traD36 proAB+ lacIqZΔM15 (Tetr)] *ΔldhA ΔadhE ΔfrdBC*	[2]
BP1	Evolved JCL166 strain functioning in minimal media	[7]
BP1 *ihfB* WT	BP1 with wild-type *ihfB* sequence	[7]
BP1 *∆ihfB*	BP1 with *ihfB* deleted	[7]
**Plasmids**	**Description or genotype**	**Source**
pEL11	PLlacO1::*atoB*_EC_-*adhE2*_CA_-*crt*_CA_-*hbd*_CA_ ColE1 *ori* Amp^r^	[3]
pIM8	PLlacO1::*ter*_TD_ ColA *ori* Kan^r^	[3]

## Supplementary Materials

The following are available online at https://www.mdpi.com/2218-1989/10/5/192/s1, Figure S1. (A) PCA score plot for metabolic profiling of JCL166 (purple) and BP1 (red) at stationary phase (24 h). The ellipse indicates 95% confidence border based on Hotelling’s T2. (B) Corresponding PCA loading plot showing metabolites (green and brown circles) that contributed to the separation of the two strains. (C) Enlarged PCA loading plot showing encircled metabolites in the positive region of PC1 in (B). Brown circles indicate the metabolites that were further discussed in the manuscript. Figure S2. Metabolite intensities of CoA-related compounds. Bar graphs show relative intensities on y-axis obtained by normalization of peak area with the internal standard. Asterisks indicate significant difference between the two strains (*: *p* < 0.05, **: *p* < 0.01). Error bars indicate standard deviation obtained from four replicates. Figure S3. Metabolite intensities of glycolysis pathway intermediates. Bar graphs show relative intensity on y-axis obtained by normalization of peak area with the internal standard. Asterisks indicate significant difference between the two strains (*: *p* < 0.05, **: *p* < 0.01). Error bars indicate standard deviation obtained from four replicates. Figure S4. Metabolite intensities of nucleotide biosynthesis-related compounds. Bar graphs show relative intensities on the y-axis obtained by normalization of peak area with the internal standard. Asterisks indicate significant difference between the two strains (**: *p* < 0.01). Error bars indicate standard deviation obtained from four replicates. Figure S5A) Growth curve of JCL166, BP1, BP1 ihfB WT and BP1 *ΔihfB* strain in 4 g/L glucose minimal medium under anaerobic conditions. (B) 1-Butanol titer (g/L) of JCL166, BP1, BP1 ihfB WT and BP1 *ΔihfB* strain from anaerobic fermentation in 4 g/L minimal medium. Error bars indicate standard deviation obtained from three biological replicates. Table S1: Data matrix subjected to PCA of BP1 and BP1 *ihfB* WT. Table S2 Optimized Multiple Reaction Monitoring (MRM) parameters for 124 metabolites targeted using IP-LC/QqQ-MS.

## References

[1] Jones D.T., Woods D.R. (1986). Acetone-Butanol Fermentation Revisited. Microbiol. Rev..

[2] Atsumi S., Cann A.F., Connor M.R., Shen C.R., Smith K.M., Brynildsen M.P., Chou K.J.Y., Hanai T., Liao J.C. (2008). Metabolic engineering of *Escherichia coli* for 1-butanol production. Metab. Eng..

[3] Shen C.R., Lan E.I., Dekishima Y., Baez A., Cho K.M., Liao J.C. (2011). Driving forces enable high-titer anaerobic 1-butanol synthesis in *Escherichia coli*. Appl. Environ. Microbiol..

[4] Nitta K., Laviña W.A., Pontrelli S., Liao J.C., Putri S.P., Fukusaki E. (2017). Orthogonal partial least squares / projections to latent structures regression-based metabolomics approach for identification of gene targets for improvement of 1-butanol production in *Escherichia coli*. J. Biosci. Bioeng..

[5] Nitta K., Laviña W.A., Pontrelli S., Liao J.C., Putri S.P., Fukusaki E. (2018). Metabolome analysis revealed the knockout of glyoxylate shunt as an effective strategy for improvement of 1-butanol production in transgenic *Escherichia coli*. J. Biosci Bioeng..

[6] Ohtake T., Pontrelli S., Laviña W.A., Liao J.C., Putri S.P., Fukusaki E. (2017). Metabolomics-driven approach to solving a CoA imbalance for improved 1-butanol production in *Escherichia coli*. Metab. Eng..

[7] Pontrelli S., Fricke R.C.B., Sakurai S.S.M., Putri S.P., Fitz-Gibbon S., Chung M., Wu H.Y., Chen Y.J., Pellegrini M., Fukusaki E. (2018). Directed strain evolution restructures metabolism for 1-butanol production in minimal media. Metab Eng..

[8] Zhang J., Greasham R. (1999). Chemically defined media for commercial fermentations. Appl. Microbiol. Biotechnol..

[9] Müller J., Beckers M., Mußmann N., Bongaerts J., Büchs J. (2018). Elucidation of auxotrophic deficiencies of *Bacillus pumilus* DSM 18097 to develop a defined minimal medium. Microb. Cell Fact..

[10] Blaby I.K., Lyons B.J., Wroclawska-Hughes E., Phillips G.C.F., Pyle T.P., Chamberlin S.G., Benner S.A., Lyons T.J., Crecy-Lagard V., Crécy E. (2012). Experimental evolution of a facultative thermophile from a mesophilic ancestor. Appl. Environ. Microbiol..

[11] Horinouchi T., Sakai A., Kotani H., Tanabe K., Furusawa C. (2017). Improvement of isopropanol tolerance of *Escherichia coli* using adaptive laboratory evolution and omics technologies. J. Biotech..

[12] Matsusako T., Toya Y., Yoshikawa K., Shimizu H. (2017). Identification of alcohol stress tolerance genes of *Synechocystis* sp. PCC 6803 using adaptive laboratory evolution. Biotechnol. Biofuels.

[13] Reyes L.H., Almario M.P., Winkler J., Orozco M.M., Kao K.C. (2012). Visualizing evolution in real time to determine the molecular mechanisms of n-butanol tolerance in *Escherichia coli*. Metab. Eng..

[14] Conrad T.M., Frazier M., Joyce A.R., Cho B.-K., Knight E.M., Lewis N.E., Landick R., Palsson B.Ø. (2010). RNA polymerase mutants found through adaptive evolution reprogram *Escherichia coli* for optimal growth in minimal media. Proc. Natl. Acad. Sci. USA.

[15] Lee D.H., Palsson B.O. (2010). Adaptive evolution of *Escherichia coli* K-12 MG1655 during growth on a nonnative carbon source, L-l,2-propanediol. Appl. Environ. Microbiol..

[16] Sonderegger M., Sauer U. (2003). Evolutionary Engineering of Saccharomyces cerevisiae for Anaerobic Growth on Xylose. Appl Environ. Microbiol..

[17] Tang J. (2011). Microbial Metabolomics. Curr Genomics..

[18] Fang X., Sastry A., Mih N., Kim D., Tan J., Yurkovich J.T., Colton J.L., Gao Y., Yang L., Palsson B.O. (2017). Global transcriptional regulatory network for Escherichia coli robustly connects gene expression to transcription factor activities. Proc. Natl. Acad. Sci. USA.

[19] Martínez-Antonio A., Collado-Vides J. (2003). Identifying global regulators in transcriptional regulatory networks in bacteria. Curr. Opin. Microbiol..

[20] Arfin S.M., Long A.D., Ito E.T., Tolleri L., Riehle M.M., Paegle E.S., Hatfield W.G. (2005). Global Gene Expression Profiling in *Escherichia coli* K12: The Effects of Integration Host Factor. J. Biol. Chem..

[21] Paul L., Blumenthal R.M. (2001). Activation from a Distance: Roles of Lrp and Integration Host Factor in Transcriptional Activation of *gltBDF*. J. Bacteriol..

[22] Chen X., Alonso A.P., Allen D.K., Reed J.L., Shachar-hill Y. (2011). Synergy between 13 C-metabolic flux analysis and flux balance analysis for understanding metabolic adaption to anaerobiosis in *E. coli*. Metab. Eng..

[23] Kargeti M., Venkatesh K.V. (2017). The effect of global transcriptional regulators on the anaerobic fermentative metabolism of *Escherichia coli*. Mol. Biosyst..

[24] Tsugawa H., Arita M., Kanazawa M., Ogiwara A., Bamba T., Fukusaki E. (2013). MRMPROBS: A data assessment and metabolite identification tool for large-scale multiple reaction monitoring based widely targeted metabolomics. Anal. Chem..

[25] Datsenko K.A., Wanner B.L. (2000). One-step inactivation of chromosomal genes in *Escherichia coli* K- 12 using PCR products. Proc. Natl. Acad. Sci. USA.

