# Supplementary material for: Metabolomics Analysis Reveals Global Metabolic Changes in the Evolved E. coli Strain with Improved Growth and 1-Butanol Production in Minimal Medium
Source: Metabolites. 2020 May 13;10(5):192. doi: 10.3390/metabo10050192 (PMC7281505; doi:10.3390/metabo10050192)
Supplement: Supplementary file 1 [file metabolites-10-00192-s001.pdf]

Supplementary Data

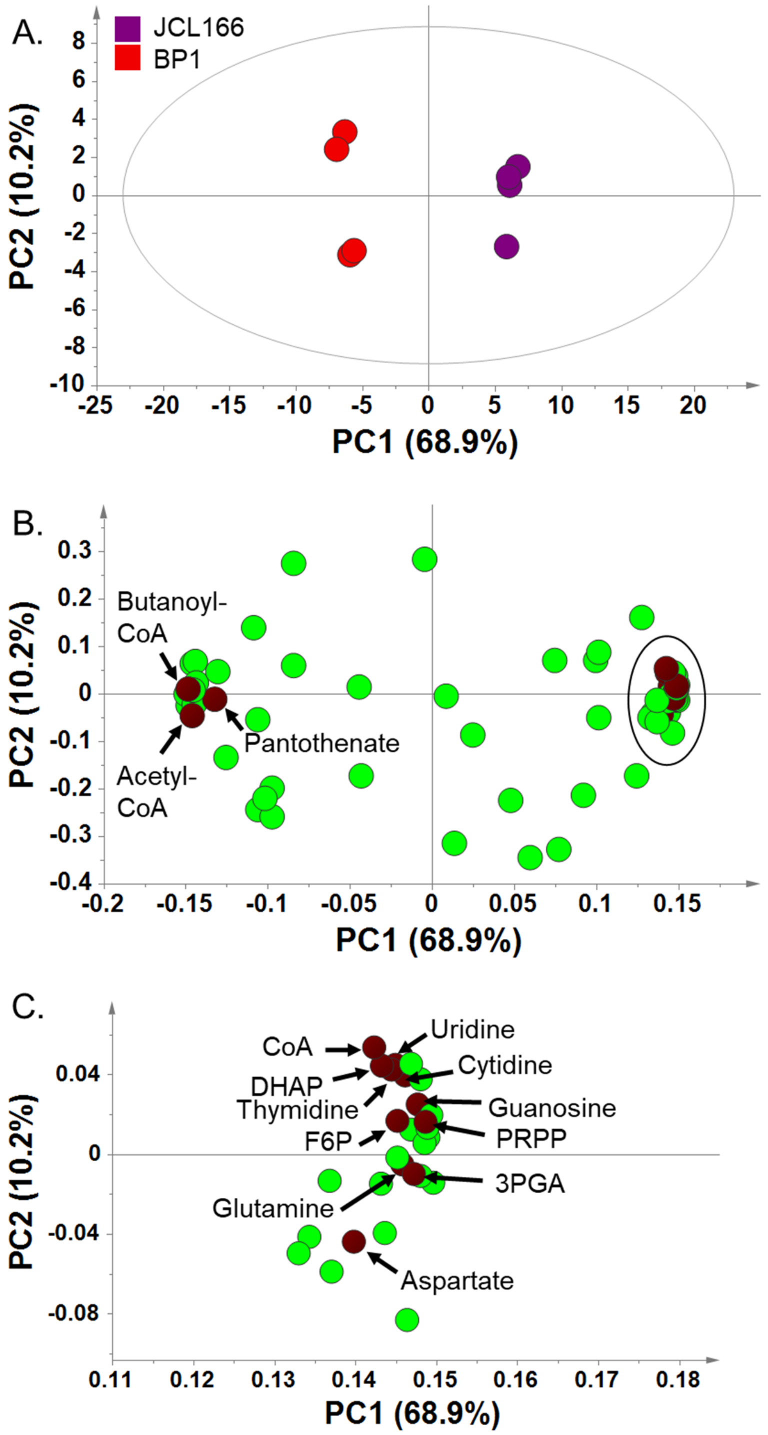

Fig. S1 **(A)** PCA score plot for metabolic profiling of JCL166 (purple) and BP1 (red) at stationary phase (24 h). The ellipse indicates 95% confidence border based on Hotelling's T^2. **(B)** Corresponding PCA loading plot showing metabolites (green and brown circles) that contributed to the separation of the two strains. **(C)** Enlarged PCA loading plot showing encircled metabolites in the positive region of PC1 in (**B**). Brown circles indicate the metabolites that were further discussed in the manuscript. Dataset derived from *Metab Eng*. *49*, 153–163, Pontrelli, S.; Fricke, R. C. B.; Sakurai, S. S. M.; Putri, S. P.; Fitz-Gibbon, S.; Chung, M.; Wu, H. Y.; Chen, Y. J.; Pellegrini, M.; Fukusaki, E.; Liao, J. C Directed strain evolution restructures metabolism for 1-butanol production in minimal media, 2018, with permission from Elsevier.

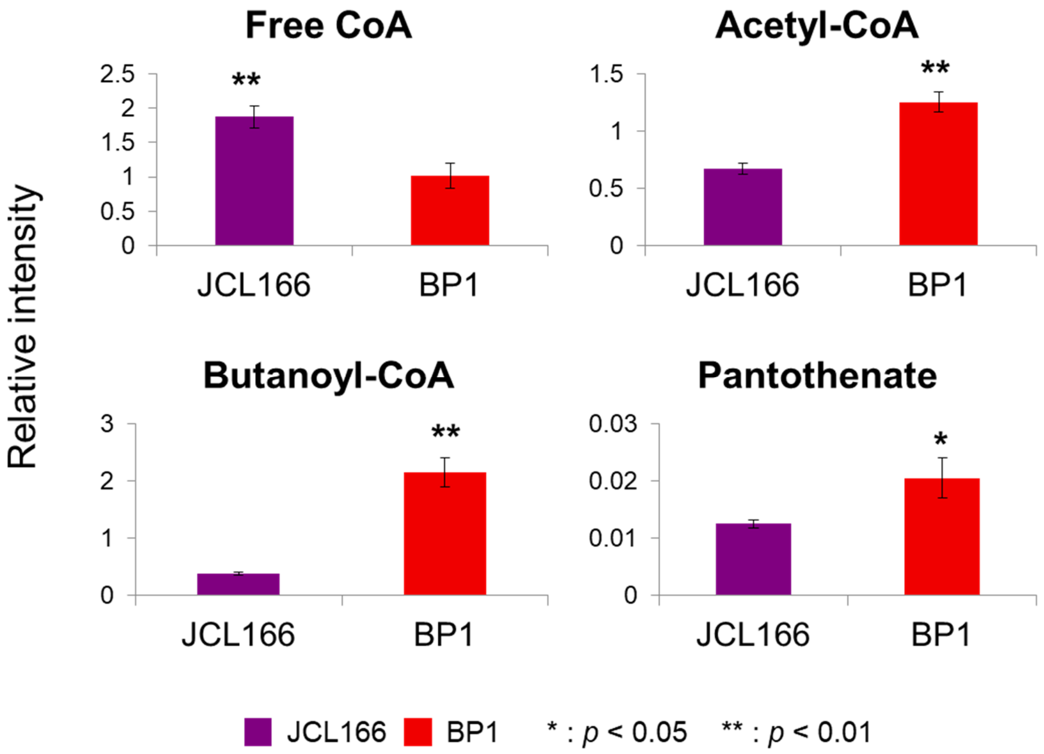

Fig. S2 Metabolite intensities of CoA-related compounds. Bar graphs show relative intensities on y-axis obtained by normalization of peak area with the internal standard. Asterisks indicate significant difference between the two strains (*: $p < 0.05$, **: $p < 0.01$). Error bars indicate standard deviation obtained from 4 replicates. Dataset derived from Metab Eng. 49, 153–163, Pontrelli, S.; Fricke, R. C. B.; Sakurai, S. S. M.; Putri, S. P.; Fitz-Gibbon, S.; Chung, M.; Wu, H. Y.; Chen, Y. J.; Pellegrini, M.; Fukusaki, E.; Liao, J. C Directed strain evolution restructures metabolism for 1-butanol production in minimal media, 2018, with permission from Elsevier.

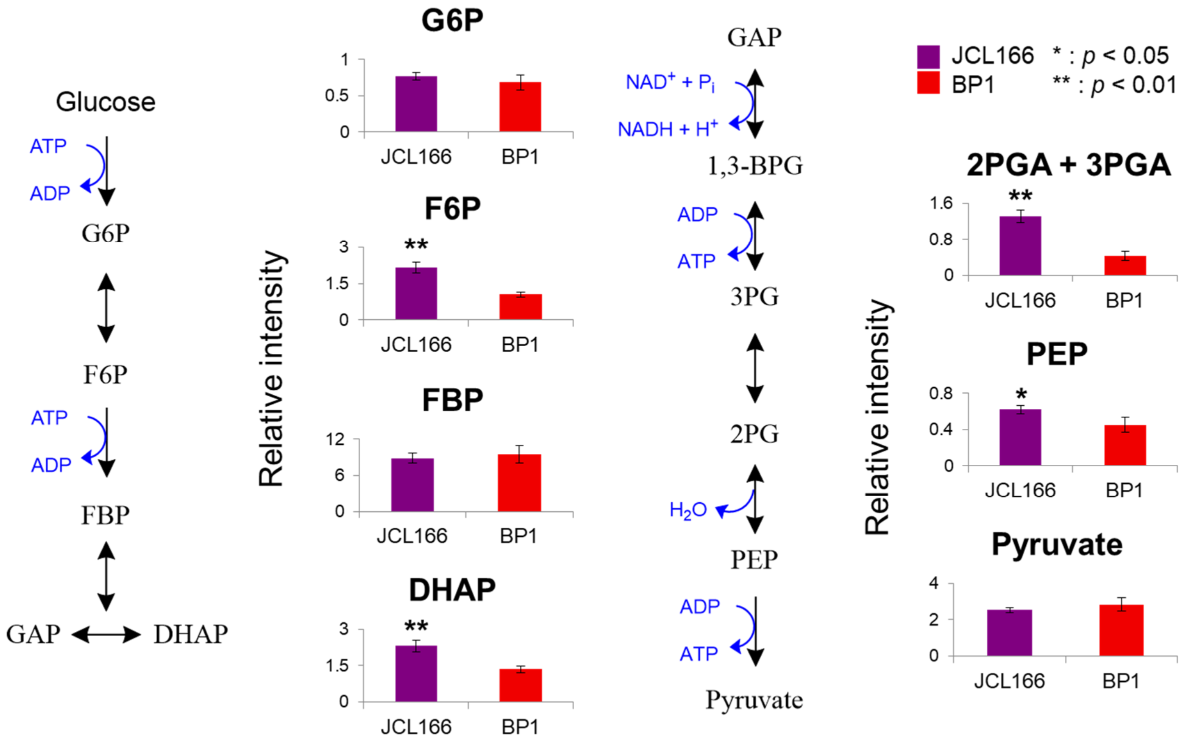

Fig. S3 Metabolite intensities of glycolysis pathway intermediates. Bar graphs show relative intensity on y-axis obtained by normalization of peak area with the internal standard. Asterisks indicate significant difference between the two strains (*: $p < 0.05$, **: $p < 0.01$). Error bars indicate standard deviation obtained from 4 replicates. Dataset derived from Metab Eng. 49, 153–163, Pontrelli, S.; Fricke, R. C. B.; Sakurai, S. S. M.; Putri, S. P.; Fitz-Gibbon, S.; Chung, M.; Wu, H. Y.; Chen, Y. J.; Pellegrini, M.; Fukusaki, E.; Liao, J. C Directed strain evolution restructures metabolism for 1-butanol production in minimal media, 2018, with permission from Elsevier.

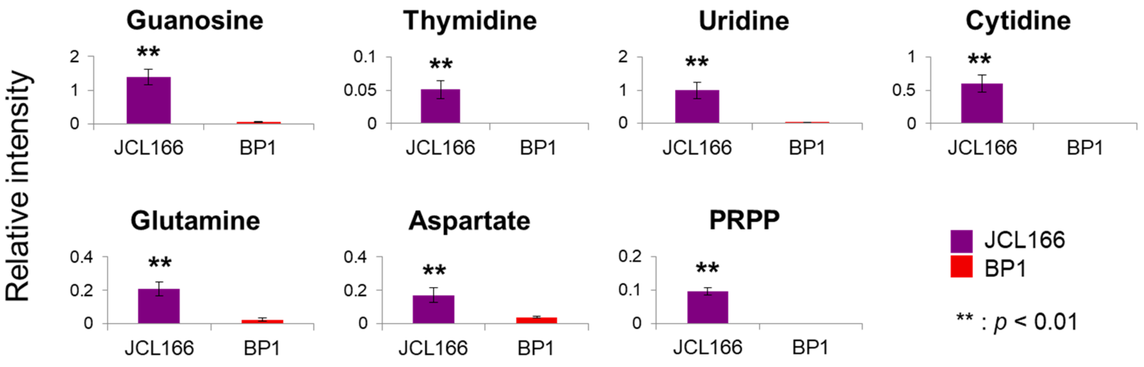

Fig. S4 Metabolite intensities of nucleotide biosynthesis-related compounds. Bar graphs show relative intensities on the y-axis obtained by normalization of peak area with the internal standard. Asterisks indicate significant difference between the two strains (**: p < 0.01). Error bars indicate standard deviation obtained from 4 replicates. Dataset derived from Metab Eng. 49, 153–163, Pontrelli, S.; Fricke, R. C. B.; Sakurai, S. S. M.; Putri, S. P.; Fitz-Gibbon, S.; Chung, M.; Wu, H. Y.; Chen, Y. J.; Pellegrini, M.; Fukusaki, E.; Liao, J. C Directed strain evolution restructures metabolism for 1-butanol production in minimal media, 2018, with permission from Elsevier.

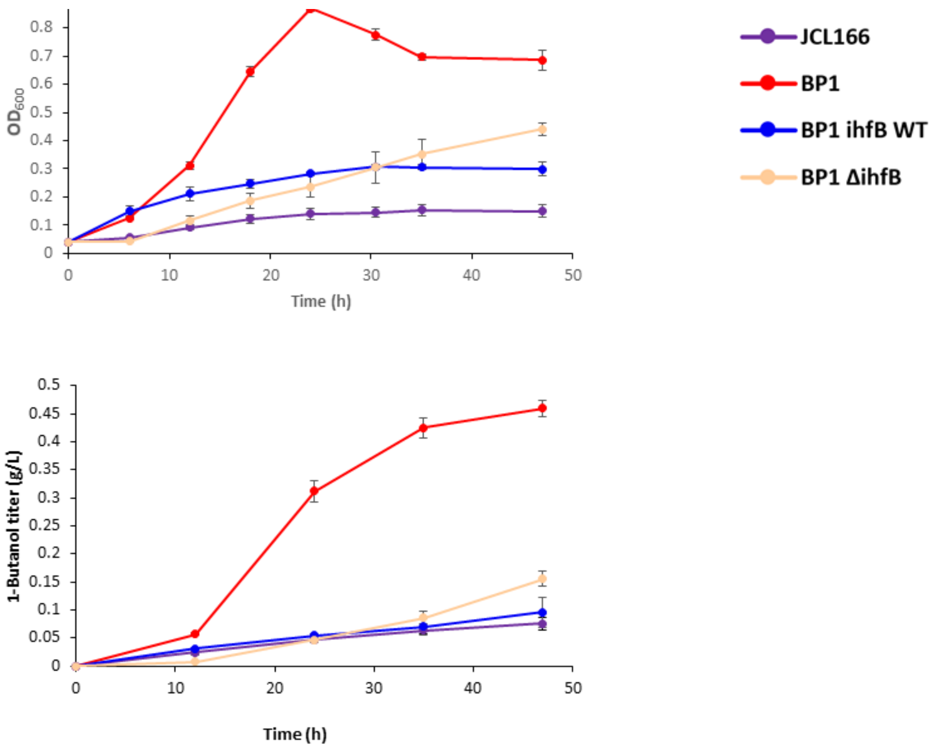

Fig. S5. A) Growth curve of JCL166, BP1, BP1 ihfB WT and BP1 *ΔihfB* strain in 4 g/L glucose minimal medium under anaerobic conditions. (B) 1-Butanol titer (g/L) of JCL166, BP1, BP1 *ihfB* WT and BP1 *ΔihfB* strain from anaerobic fermentation in 4 g/L minimal medium. Error bars indicate standard deviation obtained from 3 biological replicates.

Table. S1 Data matrix subjected to PCA of BP1 and BP1 *ihfB* WT

	**BP1-1**	**BP1-2**	**BP1-3**	**BP1 ihfB WT-1**	**BP1 ihfB WT-2**	**BP1 ihfB WT-3**
Glutamine	0.011415	0.015097	0.014592	0.093205	0.087108	0.127386
Threonine	0.031697	0.025992	0.03442	0.029334	0.033382	0.033569
Trehalose	0.639859	0.745842	0.624517	0.099901	0.114561	0.037066
Cytidine	0.029385	0.023774	0.038411	0.011868	0.042605	0.012037
Methionine	0.019481	0.018964	0.018823	0.009962	0.010608	0.009399
Tyrosine	0.019723	0.019138	0.02493	0.09738	0.103322	0.089647
Xanthine	0.001302	0.001125	0.001034	0.005578	0.008701	2.01E-05
Glutamate	0.417558	0.474426	0.459906	3.632352	3.622189	3.311581
Uridine	0.151941	0.122965	0.188447	0.053068	0.135013	0.062424
Aspartate	0.075072	0.074835	0.091359	0.107821	0.111435	0.10755
Thymine	0.002608	0.002327	0.002323	0	0	0
Guanosine	0.187952	0.154594	0.222186	0.052509	0.16883	0.060489
Adenosine	0.006349	0.005643	0.008656	0.002639	0.010743	0.003034
Phenylalanine	0.017214	0.017539	0.022789	0.074263	0.077232	0.078757
G6P	0.365081	0.450731	0.3243	1.045729	0.955416	1.07244
Sor6P	1.845134	1.931761	1.689705	1.932076	2.289274	2.050239
Tryptophan	0.025733	0.03174	0.021883	0.01003	0.007927	0.009471
R5P	0.072851	0.08062	0.072935	0.286491	0.263343	0.307214
Lactate	0.046347	0.037577	0.073839	-1E-04	-0.00104	-0.0107
F6P	0.503675	0.523952	0.404531	2.744823	2.000335	2.309836
G1P	0.458885	0.573183	0.571291	1.234093	1.441638	1.187907
a-GP	0.539839	0.617233	0.568093	0.686825	0.880521	0.633635
NAD	0.972445	0.938175	0.955549	0.799174	0.852416	0.89983
Orotate	7.450467	6.601834	6.713027	14.16377	13.70175	12.33199
Ru5P+Xu5P	0.478489	0.618823	0.451125	1.929128	2.160991	2.230475
CMP	0.079758	0.074551	0.082908	0.165169	0.217903	0.162784
Pyruvate	0.61319	0.548166	0.54433	1.598509	1.562846	1.386458
R1P	0.117788	0.096708	0.16779	0.13415	0.233655	0.12611
UMP	0.254697	0.250938	0.337378	0.351355	0.427584	0.284544
AICAR	0.126201	0.125124	0.132756	0.223528	0.210611	0.243252
GMP	0.088299	0.09199	0.125823	0.122402	0.166652	0.080409
IMP	0.052649	0.064323	0.062451	0.082421	0.084012	0.086397
DHAP	2.240666	2.733841	2.596046	4.14188	5.174906	5.083663
TMP	0.164723	0.127206	0.157776	0.197575	0.17669	0.192771
AMP	0.847916	0.87703	1.112971	1.112288	2.000808	0.940613

Pantothenate	0.026509	0.023808	0.027788	0.302301	0.302048	0.345557
Nicotinate	0.053194	0.047007	0.056386	0.057667	0.054691	0.053059
Succinate	0.869114	0.731885	0.798384	0.471794	0.510606	0.385084
Malate	1.221566	1.141971	1.373237	1.734686	1.986808	1.662247
UDP-Glc	2.065808	1.930819	2.023611	3.500157	3.398332	3.838185
XMP	0.024917	0.032207	0.029272	0.015733	0.019882	0.019424
CDP	0.147474	0.129107	0.159531	0.151969	0.207305	0.131024
Acetyl-P	0.100199	0.182934	0.126369	0.141259	0.064364	0.038319
ADP-Glc	0.008999	0.008919	0.008691	0.029148	0.022721	0.015708
GDP	0.099026	0.084387	0.10115	0.214735	0.256511	0.207023
6PGA	0.538831	0.632335	0.560526	0.909202	0.935955	0.971294
2PGA+3PGA	0.968114	1.156164	0.964917	1.842556	1.831351	1.986348
NADP	0.252748	0.275205	0.276938	0.342415	0.362266	0.372036
ADP	0.099633	0.099713	0.103874	0.241188	0.325915	0.206756
SBP	0.133009	0.137151	0.134011	0.188249	0.202839	0.259061
FBP	5.71904	5.861737	5.505976	3.59836	3.75076	4.819577
PEP	0.679177	0.677917	0.899807	0.805611	1.308833	0.818204
Iso-/citrate	4.2306	4.648352	2.9252	9.715756	8.822293	9.079553
2-Isopropylmalate	0.76358	0.737427	0.734253	0.842359	0.91805	0.92632
GTP	0.486168	0.489364	0.405974	1.265197	1.222387	1.336522
CTP	0.420632	0.397884	0.36163	0.596392	0.577656	0.52436
UTP	2.461343	2.437211	2.175919	1.764168	1.855995	1.454997
ATP	0.840911	0.805222	0.662249	2.127732	1.863682	2.002572
FAD	0.051862	0.038811	0.041675	0.038248	0.031503	0.028898
PRPP	0.25919	0.256899	0.264702	0.627935	0.549981	0.864633
CoA	1.437538	1.301405	1.239883	1.911884	2.316226	1.858471
3HB-CoA	0.007985	0.007177	0.011098	0.035834	0.030752	0.042835
IPP,DMAPP	0.032551	0.034553	0.031134	0.07052	0.078928	0.068909
Malonyl-CoA	0.008486	0.004875	0.010104	0.033497	0.027845	0.044771
Acetyl-CoA	1.476448	1.456737	1.445948	1.790139	1.558383	1.876677
Butanoyl-CoA	1.559542	1.530566	1.525437	0.2649	0.245996	0.333833

Table. S2 Optimized Multiple Reaction Monitoring (MRM) parameters for 124 metabolites targeted using IP-LC/QqQ-MS. *m/z* (1): Precursor ion *m/z*; *m/z* (2): Product ion *m/z*; Ret. Time: Retention Time

**Metabolite**	***m/z* (1)**	***m/z* (2)**	**Ret. Time**	**Target Q1 Pre Bias (V)**	**Target Collision Energy (V)**	**Target Q3 Pre Bias (V)**
**Arginine**	173.05	131.05	1.272	13	15	25
**Lysine**	145.1	97.05	1.275	10	13	18
**Histidine**	154.05	93	1.277	12	21	16
**4-Aminobutanoate**	162.05	102	1.465	11	8	18
**Serine**	104.05	74.1	1.653	12	16	13
**Asparagine**	131.05	113.05	1.659	10	15	21
**Glutamine**	145.1	127.05	1.691	12	18	18

**Threonine**	118.05	74.05	1.702	21	16	26
**Hydroxyproline**	190.05	130.05	1.709	13	10	23
**Hexose**	179.05	89	1.743	13	19	15
**2-Aminobutanoate**	162.05	102	1.817	11	8	18
**Cysteine**	239.05	120.1	1.825	11	13	21
**Trehalose**	341.05	89.1	1.885	27	23	16
**Proline**	174.05	114	1.898	11	10	20
**Sucrose**	341.05	89.1	2.074	27	23	16
**Valine**	176.05	116.05	2.201	12	10	20
**Cytidine**	302.05	242	2.337	24	10	19
**Pyridoxamine-5P**	247.05	230	2.453	17	11	23
**Methionine**	148.05	47.05	2.767	11	14	16
**Guanine**	150.05	133.05	3.126	11	21	23
**Hypoxanthine**	135.05	92	3.546	13	28	14
**Tyrosine**	180.05	163.05	3.554	12	18	18
**Adenine**	134.1	107.05	3.589	14	22	19
**Isoleucine**	190.05	130.05	3.638	13	10	23
**Leucine**	190.05	130.05	3.963	13	10	23
**Xanthine**	151.05	108.05	3.978	10	18	18
**Glutamate**	146.05	102.05	4.284	11	15	18
**Uridine**	243.05	110.05	4.325	19	17	20
**Aspartate**	132.05	88.05	4.426	10	14	15
**Inosine**	267.05	135.05	4.566	21	23	25
**Thymine**	125.05	42	4.575	10	18	14
**Guanosine**	282.1	150.05	4.605	23	21	29
**Urate**	167.1	124.05	4.699	11	15	21
**Shimikate**	173.05	93	4.854	11	17	16
**Adenosine**	266.1	134.05	4.975	18	25	23
**Glycerate**	105.05	75.05	5.097	12	13	26
**Thymidine**	301.1	241	5.151	23	10	18
**Phenylalanine**	164.05	147.05	5.208	13	18	27
**Glycolate**	75.05	47.05	5.335	16	13	16
**Glyoxylate**	73	73	5.754	15	5	15
**G6P**	259.05	97	6.303	20	17	17
**Disaccharide-P**	421.1	79.05	6.372	29	40	27
**Mn6P**	259.05	97	6.535	18	17	17
**Sor6P**	261.05	97	6.593	19	23	16
**Pyroglutamate**	188.05	128	6.618	22	12	20
**Tryptophan**	203.1	116.05	6.675	13	18	19
**R5P**	229.05	97	6.691	18	13	18
**SSA**	101.05	57	6.697	20	13	19
**Lactate**	89.05	43	6.779	20	14	15
**S7P**	289.1	97	6.803	20	21	16
**F6P**	259.05	97	6.823	20	17	17
**Ara5P**	229.05	97	6.982	16	13	17

**G1P**	259.05	79.05	7.062	20	28	27
**a-GP**	171.05	79.05	7.063	13	18	13
**TPP**	424.1	302.05	7.103	30	16	20
**NAD**	662.1	540.1	7.188	26	18	26
**GAP**	169.05	97	7.288	13	12	17
**Orotate**	155.05	111.05	7.301	12	14	20
**Ru5P**	229.05	97	7.459	18	13	18
**CMP**	322.1	79.05	7.488	25	28	14
**b-GP**	171.05	79.05	7.597	13	18	13
**MEP**	215.05	79.05	7.623	24	27	29
**F1P**	259.05	97	7.651	20	17	17
**Pyruvate**	87.05	43	7.782	10	11	14
**R1P**	229.05	79.05	7.812	16	25	27
**UMP**	323.1	79.05	7.951	26	36	13
**AICAR**	337.1	79.05	8.001	12	37	26
**GMP**	362.1	79.05	8.031	27	26	13
**IMP**	347.05	79.05	8.049	25	40	28
**DHAP**	169.05	97	8.085	13	12	17
**TMP**	321.1	195.05	8.572	25	20	22
**AMP**	346.1	79.05	8.618	14	38	13
**Pantothenate**	218.05	88	8.814	17	17	15
**Nicotinate**	122.05	78	8.818	13	16	13
**cAMP**	328.1	134.05	9.143	26	27	25
**Succinate**	117.05	73	9.568	13	15	12
**Carbamoyl-P**	140.05	79.05	9.569	10	22	26
**Glutathione**	306.05	143.05	9.571	16	20	26
**Malate**	133.05	115	9.848	10	17	21
**UDP-Glc**	565.05	323.05	9.878	22	27	15
**XMP**	363.1	211.05	9.961	27	20	21
**CDP**	402.1	79.05	10.021	16	42	14
**Acetyl-P**	139	79.05	10.045	27	14	30
**2OG**	145.1	101.05	10.046	15	10	17
**ADP-Glc**	588.05	346.05	10.069	24	23	24
**Fumarate**	115.05	71	10.128	13	10	12
**GDP**	442.1	79.05	10.131	18	45	13
**6PGA**	275.05	177.05	10.154	19	16	30
**UDP**	403.1	159	10.176	16	28	29
**3PGA**	185.05	97	10.181	14	16	17
**KDPG**	257.05	97	10.215	10	18	17
**Shikimate-3P**	253.05	97	10.224	18	13	17
**NADH**	664.1	79.05	10.238	24	57	13
**NADP**	742.1	620.1	10.329	26	18	30
**ADP**	426.1	79.05	10.353	17	46	13
**SBP**	369.1	97	10.374	14	27	17
**Citrate**	191.05	87	10.399	13	18	14

**FBP**	339.05	97	10.423	26	18	17
**PEP**	167.05	79.05	10.477	17	15	27
**RuBP**	309.05	97	10.482	24	18	17
**HMBPP**	261.05	79.05	10.518	19	23	28
**Isocitrate**	191.05	73	10.568	13	22	26
**FMN**	455.1	97	10.593	18	27	17
**2-Isopropylmalate**	175.05	115.05	10.603	13	18	21
**GTP**	522.1	159	10.706	20	33	29
**CTP**	482.1	159	10.729	19	36	29
**UTP**	483.1	159	10.759	19	36	29
**ATP**	506.1	159	10.801	20	40	29
**BPG**	265.05	167.05	10.845	20	18	29
**Indol-Ac**	174.05	130.05	10.848	11	13	24
**FAD**	784.1	346.1	10.863	20	37	23
**PRPP**	389.1	177.05	10.948	28	21	30
**NADPH**	744.1	159.05	10.976	26	49	30
**PQQ**	329.1	241.05	11.051	12	15	24
**(+)-10-Camphorsulfonic acid**	231.1	80	11.055	10	32	30
**CoA**	766.1	408.1	11.105	20	30	27
**3HB-CoA**	852.1	772.1	11.109	32	41	34
**IPP, DMAPP**	245.05	79.05	11.123	17	27	27
**Malonyl-CoA**	852.1	808.1	11.125	26	27	40
**Acetyl-CoA**	808.1	408.1	11.147	20	37	28
**HMG-CoA**	910.1	408.1	11.148	34	48	26
**Succinyl-CoA**	866.1	408.1	11.149	30	44	25
**Crotonyl-CoA**	834.1	408.1	11.299	30	36	26
**Butanoyl-CoA**	836.1	408.1	11.373	31	37	26
